# Virologic outcomes of switching to dolutegravir functional mono- or dual therapy with a non-cytosine nucleoside analog: a retrospective study of treatment-experienced, patients living with HIV

**DOI:** 10.1186/s12981-021-00352-0

**Published:** 2021-05-03

**Authors:** Charlotte-Paige Rolle, Vu Nguyen, Federico Hinestrosa, Edwin DeJesus

**Affiliations:** 1 Orlando Immunology Center, 1707 North Mills Avenue, Orlando, FL 32803 USA; 2 Department of Global Health, Emory University Rollins School of Public Health, 1518 Clifton Road NE, Atlanta, GA 30322 USA; 3 University of Central Florida College of Medicine, 6850 Lake Nona Blvd., Orlando, FL 32827 USA

**Keywords:** Dolutegravir, HIV drug resistance, Monotherapy, Dual therapy

## Abstract

**Background:**

Dolutegravir (DTG) monotherapy results in virologic failure and the development of DTG resistance. Here, we evaluated virologic outcomes of patients switched to DTG functional mono- or dual therapy with a non-cytosine nucleoside analog (NA).

**Methods:**

This retrospective, single center study included treatment-experienced patients switched to regimens containing ≥ 2 antiretrovirals between 8/13/13–11/22/14 who were later found to be on DTG functional mono- or dual therapy with a non-cytosine NA based on historical genotypes. Eligible patients were either suppressed or viremic at baseline and had ≥ 2 HIV-1 RNA measurements at least 4 weeks apart following switch. Demographics, laboratory values and clinical parameters were extracted from the charts of all eligible patients during study treatment until 12/31/2018 and were summarized using descriptive statistics. The primary endpoint was the proportion of patients with HIV-1 RNA < 50 copies/mL following switch.

**Results:**

Of 70 patients switched to DTG functional mono- or dual therapy, 39 were eligible; 19 (49%) were on DTG functional monotherapy and 20 (51%) were on DTG functional dual therapy with a non-cytosine NA. Historical genotypes indicated that all had an M184V/I, and 23 (59%) had an M184V/I and ≥ 1 additional NA mutation. The median duration of follow-up on study treatment was 50 weeks (range 12–244). Following switch, 32/39 (82%) patients achieved or maintained an HIV-1 RNA < 50 copies/mL and 7 (18%) had persistent HIV-1 RNA ≥ 50 copies/mL. Five viremic patients were found to be on functional dual therapy with DTG plus a non-cytosine NA and 2 were on DTG functional monotherapy. Five of these patients had post-switch genotypes ordered as a part of routine clinical care and there was no evidence of treatment-emergent resistance. Five were switched to a different DTG-containing regimen and achieved HIV-1 RNA < 50 copies/mL, 1 was switched to a non-DTG containing regimen and achieved HIV-1 RNA < 50 copies/mL and 1 was lost-to-follow up at week 36.

**Conclusions:**

In this real-world cohort, the majority of whom had virus with the M184V/I and ≥ 1 additional NA mutation, switching to DTG functional mono-or dual therapy with a non-cytosine NA resulted in persistent HIV-1 RNA ≥ 50 copies/mL in 18%. None with post-switch genotypes developed treatment-emergent resistance.

## Introduction

Phase 3 clinical trials have demonstrated the efficacy of 2-drug dolutegravir (DTG) containing regimens (DCRs) in both treatment-naïve and -experienced adults. In the GEMINI 1&2 studies conducted in treatment-naïve adults, DTG/lamivudine (3TC) was found to be non-inferior to DTG plus tenofovir disoproxil fumarate (TDF)/emtricitabine (FTC) with 82% on DTG/3TC versus 84% on DTG plus TDF/FTC achieving virologic suppression through Week 144 [1]. In the TANGO study conducted in treatment-experienced, virologically suppressed adults on a baseline tenofovir alafenamide (TAF)-containing regimen, switching to DTG/3TC was found to be non-inferior to staying on baseline regimen with virologic failure (VF) observed in < 1% of those switched to DTG/3TC vs. 1% of those remaining on a TAF-based regimen at Week 96 [2]. In both the GEMINI and TANGO studies, patients with baseline nucleoside reverse transcriptase inhibitor (NRTI) resistance include those with M184V/I and integrase strand transfer inhibitor (INSTI) resistance were excluded [1, 2]. In the GEMINI studies, there were 12 patients in the DTG/3TC arm versus 9 patients in the comparator arm with confirmed virologic withdrawal (CVW) at Week 144, however none developed treatment-emergent resistance [1]. In the TANGO study, there were no confirmed virologic withdrawals in the DTG/3TC arm through Week 96, hence no patients were evaluated for treatment-emergent resistance. There were 3 CVWs among those continuing their TAF-based regimen and no treatment-emergent resistance was observed [2].

In the SWORD 1&2 studies conducted among treatment-experienced, virologically suppressed adults on a stable regimen consisting of two NRTIs and a third agent, switching to DTG/rilpivirine (RPV) was non-inferior to remaining on baseline regimen through Week 52 with VF observed in < 1% switching to DTG/RPV versus < 1% remaining on baseline regimen [3]. At Week 52, all patients in the study were switched to DTG/RPV and VF occurred in 3% in the “early-switch” group and 2% in the “late-switch” group through Week 148 [4]. Among 11 CVWs, 6 developed resistance to RPV, and none developed resistance to DTG [4].

Other clinical trials of stably suppressed patients switched to DTG monotherapy revealed that a high proportion of VFs developed DTG resistance [5–7]. This suggests that DTG monotherapy and certain 2-drug DCRs may be ‘less forgiving’ regarding the risk of resistance development in the setting of VF. This has important implications in real-world settings where a multitude of factors including patient characteristics, disease characteristics and other social and clinical barriers may contribute to higher rates of VF than is observed in randomized clinical trials [8]. Given the increased use of DCRs globally with several low- and middle-income countries (LMICs) switching to the use of DTG-based therapy as first-and-second line regimens in response to increasing non-nucleoside reverse transcriptase inhibitor (NNRTI) resistance [9–12], real-world studies to evaluate non-traditional combinations of DTG with other antiretroviral (ARV) drugs would be useful to provide further information about the efficacy and barrier to resistance of this second-generation integrase strand transfer inhibitor (INSTI).

This is particularly relevant in LMICs where 50% of patients do not have access to routine viral load monitoring and genotypic resistance tests in the setting of VF [10]. Prior reports have demonstrated a high prevalence of nucleoside reverse transcriptase inhibitor (NRTI) resistance and M184V/I mutation among treatment-experienced adults in LMICs [12], especially among those failing first-line NRTI plus NNRTI therapy and those failing second-line NRTI plus protease inhibitor (PI) therapy [13, 14]. Though studies from high-income settings have demonstrated high suppression rates with the use of DTG-based regimens in the setting of pre-existing NRTI resistance including among those with an active or archived M184V/I mutation [15–18], little is currently known about the efficacy of switching to DTG-based functional mono or dual therapy in LMICs and other settings where the availability of cumulative resistance tests and complete ARV drug histories may be more limited.

Here, we present virologic outcomes of patients switched to DCRs with ≥ 2 ARV drugs who were subsequently found to be on DTG functional mono-or dual therapy with a non-cytosine nucleoside analog (NA).

## Methods

This was a retrospective study to describe virologic outcomes of patients switched to DTG functional mono- or dual therapy with a non-cytosine NA. Eligible patients included all patients living with HIV (PLWH) seen at the Orlando Immunology Center between 8/13/13–11/22/14 switched to once-daily DCRs with ≥ 2 ARV drugs whose historical genotypes predicted that DTG alone or in combination with a non-cytosine NA were the only fully active ARV drugs in the regimen. Historical genotypes were discovered and reviewed during a routine clinic audit conducted in December of 2014, and due to the retrospective nature of the study, it is unknown whether they were available or assessed prior to switch. Historical genotypes included assays utilizing Sanger sequencing of protease (PR), reverse transcriptase (RT) and integrase and were performed 1–9 years (median 7) prior to switch. The Stanford HIV drug resistance database (HIVdb) algorithm was used to interpret drug resistance mutations from historical genotypes and make predictions about the activity of ARV drugs in the regimen [19]. ARV drugs with a HIVdb interpretation of “susceptible” were considered to be “fully active” whereas ARV drugs with the following interpretations: potential low-level resistance, and low- medium-or high-level resistance were not considered fully active. Eligible patients were either suppressed or viremic at baseline, must have attended at least two clinic visits during the study period and had a minimum of two HIV-1 RNA measurements at least 4 weeks apart following switch. Patients with the following baseline mutations associated with reduced susceptibility to DTG: T66K, E92Q, G118R, E138 K/A/T, G140 S/A/C, Q148 H/R/K, N155H and R263K [20] were excluded. Informed consent was waived due to the retrospective nature of the study which utilized data collected as a part of routine clinical care.

Demographics, laboratory values and clinical parameters were extracted from the charts of eligible patients during DCR treatment until 12/31/2018. Descriptions of adherence were summarized based on clinician documentation in the medical record. “Post-switch” genotypes obtained as a part of routine clinical care included genotypic assays utilizing both Sanger and Next-Generation Sequencing (NGS) of PR, RT, and integrase. Similarly, the HIVdb algorithm was used to interpret post-switch genotype results and predict whether any treatment-emergent drug resistance was present [19]. The primary endpoint was the proportion of patients achieving or maintaining an HIV-1 RNA < 50 copies/mL following switch. Persistent viremia was defined as an HIV-1 RNA ≥ 50 copies/mL throughout the study period. Descriptive statistics were calculated for participant baseline demographic and clinical characteristics, virologic outcomes, and discontinuations throughout the study. The Sterling Institutional Review Board (IRB) determined that the study met IRB exemption criteria based on the retrospective nature of the study (Sterling IRB ID 7115).

## Results

**Fig. 1:**
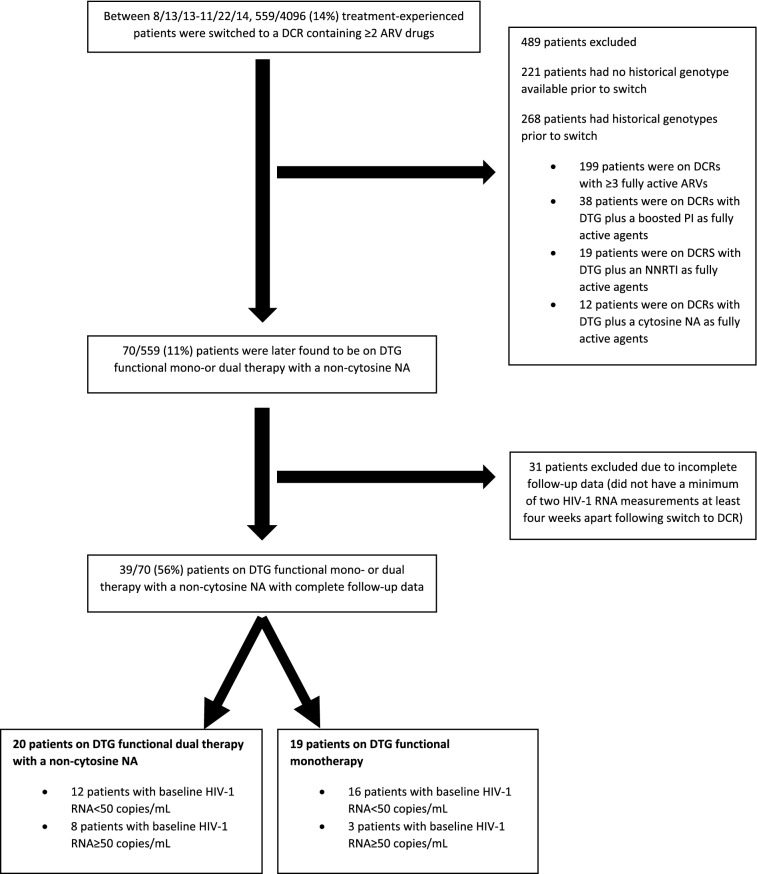
Study flowchart

During the study period, 559 treatment-experienced patients were switched to a DCR consisting of ≥ 2 ARV drugs. Based on available historical genotypes, 70/559 (11%) were found to be on DTG functional mono-or dual therapy with a non-cytosine NA, however only 39/70 (56%) were eligible and had complete follow-up data (≥ two HIV-1 RNA measurements at least 4 weeks apart following switch) to be included in the study (Fig. 1). Eighteen/39 (46%) were switched DTG plus ABC/3TC, 14/39 (36%) were switched to DTG plus TDF/FTC and 7/39 (18%) were switched to other DCRs (Table 1). Of these, 19 (3%) were found to be on DTG functional monotherapy and 20 (3%) were found to be on functional dual therapy with DTG plus a non-cytosine NA (9 on DTG/TDF and 11 on DTG/abacavir (ABC) (Fig. 1, Table 1).

**Table 1:** Baseline demographic and clinical characteristics

Characteristic	N = 39
Median age (range)	53 (40–74)
Sex	
Male, n (%)	36 (92)
Female, n (%)	3 (8)
Race/ethnicity	
Caucasian, n (%)	31 (80)
Black, n (%)	4 (10)
Hispanic, n (%)	4 (10)
Other, n (%)	0
Median BMI (range)	25.9 (17.3–36.4)
Baseline HIV viral load	
< 50 copies/mL, n (%)	28 (72)
50–200 copies/mL, n (%)	6 (15)
201–399 copies/mL, n (%)	2 (5)
≥ 400 copies/mL, n (%)	3 (8)
Median baseline CD4^+^ cell count, cells/mm^3^ (range)	564 (92–1217)
HIV disease status	
Asymptomatic, n (%)	32 (82)
Symptomatic, n (%)	7 (18)
AIDS, n (%)	0
Regimen prior to switching to DCR	
Dual NRTI + PI	15 (38)
Dual NRTI + RAL	6 (15)
Dual NRTI + NNRTI	5 (13)
TDF/FTCEVG/c	2 (5)
DRV/r + RAL	1 (3)
Regimens containing ≥ 3 ARV classes	10 (26)
Prior ARV experience	
> 2 NRTIs, n (%)	25 (64)
1 INSTI, n (%)	22 (56)
> 1 INSTI, n (%)	2 (5)
Median number of ARV regimens prior to DCR (range)	4 (1–11)
Baseline DCR	
DTG + ABC/3TC	18 (46)
DTG + TDF/FTC	14 (36)
DTG + DRV/r	2 (5)
DTG + TDF/FTC/RPV + DRV/r	2 (5)
DTG + RPV	1 (3)
DTG/ABC/3TC + TDF	1 (3)
DTG + TDF/FTC + RPV	1 (3)
Active ARV drugs in DCR based on historical genotypes	
DTG functional monotherapy, n (%)	19 (49)
DTG + non-cytosine nucleoside analog, n (%)	20 (51)
DTG + TDF, n (%)	9 (23)
DTG + ABC, n (%)	11 (28)
Historical genotypic resistance	
Overall group, n	39
Pattern of NRTI RAMs	
M184V/I alone, n (%)	16 (41)
M184V/I + 1 NRTI RAM, n (%)	5 (13)
M184V/I + > 1 NRTI RAM, n (%)	18 (46)
Number of RAMS	
NRTI RAMs, median (range)	2 (0–9)
NNRTI RAMs, median (range)	2 (0–6)
PI RAMs, median (range)	4 (0–14)
INSTI RAMs, median (range)	0 (0–1)
DTG functional monotherapy, n (%)	19 (49)
NRTI RAMs, median (range)	5 (2–9)
INSTI RAMs, median (range)	0 (0–1)
DTG + non-cytosine nucleoside analog, n (%)	20 (51)
NRTI RAMs, median (range)	1 (0–8)
INSTI RAMs, median (range)	0 (0–1)
Predicted ARV drug resistance based on historical genotype	
TDF resistance, n (%)	12 (31)
ABC resistance, n (%)	21 (54)
3TC or FTC resistance, n (%)	39 (100)
RPV resistance, n (%)	4 (10)
DRV resistance, n (%)	4(10)

*BMI* body mass index, *ARV* antiretroviral, *NRTI* nucleoside reverse transcriptase inhibitor, *NNRTI* non-nucleoside reverse transcriptase inhibitor, *PI* protease inhibitor, *RAL* raltegravir, *EVG/c* elvitegravir/cobicistat, *DRV/r* darunavir/norvir, *INSTI* integrase strand transfer inhibitor, *DCR* DTG containing regimen, *DTG* dolutegravir, *TDF* tenofovir disoproxil fumarate, *ABC* abacavir, *3TC* lamivudine, *RPV* rilpivirine, *RAM* resistance associated mutation

The median age (range) of patients was 53 (40–74) years, 28 (72%) had baseline HIV-1 RNA < 50 copies/mL, and 11 (28%) had baseline HIV-1 RNA ≥ 50 copies/mL (Table 1). The median number (range) of ARV regimens prior to switch was 4 (1–11). None of the patients had previously used DTG. ARV regimens prior to switching to the DCR included a dual NRTI plus boosted PI in 15 (38%), a dual NRTI plus raltegravir (RAL) in 6 (15%), a dual NRTI plus NNRTI in 5 (13%), TDF/FTC/elvitegravir/cobicistat (EVG/c) in 2 (5%), darunavir/norvir (DRV/r) plus RAL in 1 (3%) and 10 (26%) were switched from regimens containing ≥ 3 ARV drug classes. A review of complete ARV drug histories revealed that 25 (64%) had previously used ≥ 2 nucleoside reverse transcriptase inhibitors (NRTIs), and 24 (62%) had previously used one of the first-generation INSTIs, RAL or EVG/c. All patients had virus with the M184V/I however 23 (59%) had additional NRTI resistance associated mutations (RAMs). Interpretation of historical RAMs using the Stanford HIVdb revealed that all patients had FTC/3TC resistance, 21 (54%) had ABC resistance, 12 (31%) had TDF resistance, 4 (10%) had RPV resistance and 4 (10%) had DRV resistance. Reasons for regimen switch included reducing pill burden (17/39), patient co-morbidities (9/39), persistent viremia (4/39), side effect concerns from prior regimen (2/39) and 7/39 had no reason documented (Table 1).

**Fig. 2:**
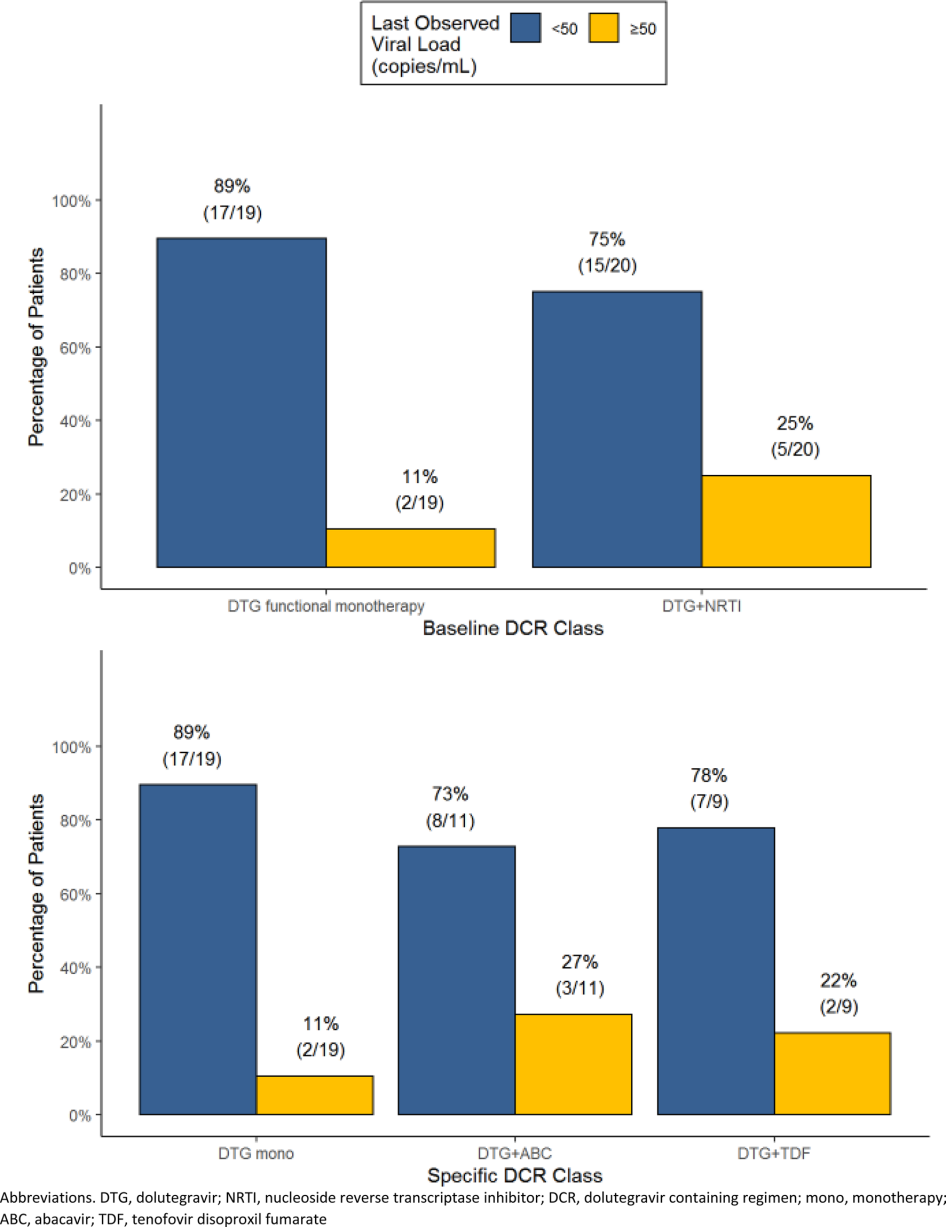
Subgroup analysis of virologic outcomes throughout the study period. *DTG* dolutegravir, *NRTI* nucleoside reverse transcriptase inhibitor, *DCR* dolutegravir containing regimen, *mono* monotherapy, *ABC* abacavir, *TDF* tenofovir disoproxil fumarate

Following switch, 32/39 (82%) patients achieved or maintained an HIV-1 RNA < 50 copies/mL, and 7 (18%) patients experienced persistent HIV-1 RNA ≥ 50 copies/mL. The median duration (range) of follow-up was 50 weeks (12–244). Eighty-nine percent (17/19) of patients treated with functional DTG monotherapy had HIV-1 RNA < 50 copies/mL compared to 75% (15/20) of those treated with functional dual therapy with DTG and a non-cytosine NA (7 on DTG/TDF and 8 on DTG/ABC) (Fig. 2).

Among the 32 patients who achieved an HIV-1 RNA < 50 copies/mL on the study DCR, 7/32 (22%) had an HIV-1 RNA ≥ 50 copies/mL at baseline whereas 25/32 (78%) had an HIV-1 RNA < 50 copies/mL at baseline. All of these patients had a baseline M184V/I and 21/32 (66%) had ≥ 1 additional NRTI mutation. Twelve (37.5%) were switched from a dual NRTI plus boosted PI, 6 (19%) were switched from a dual NRTI + RAL, 4 (12.5%) were switched from a dual NRTI + NNRTI, 1 (3%) was switched from TDF/FTC/EVG/c, 1 (3%) was switched from DRV/r plus RAL and 8 (25%) were switched from ARV regimens containing ≥ 3 ARV drug classes. Medical records review revealed that “100% adherence” was documented on the study DCR for all 32 patients. After the study period ended, 7 patients on functional DTG dual therapy with a non-cytosine NA continued the study DCR, 2 patients on functional DTG dual therapy discontinued the study DCR but switched to a different DCR and 6 patients on functional DTG dual therapy discontinued the study DCR and switched to a regimen that did not contain DTG. Of those on functional DTG monotherapy, 10 continued the study DCR after the study period ended, 3 discontinued the study DCR but switched to a different DCR and 4 discontinued the study DCR and switched to a regimen that did not contain DTG. All patients who discontinued the study DCR (15/32) were virally suppressed (HIV-1 RNA < 50 copies/mL) at the time of switch and reasons for switch included resistance noted on historical genotypes in 12/15 and side effects in 3/15 (1 patient with rash, 1 with dizziness and 1 with insomnia).

Of those with persistent HIV-1 RNA ≥ 50 copies/mL during the study, 2 were on DTG functional monotherapy, and 5 were on functional dual therapy (2 on DTG/TDF and 3 on DTG/ABC) (Fig. 2, Table 2). Both functional monotherapy patients had a baseline HIV-1 RNA ≥ 50 copies/mL, one was switched from ABC/3TC/DRV/r to DTG/RPV (Table 2, Patient 5) and historical genotypic testing revealed an M184V/I and E138E/K which predicted that DTG was the only fully active agent in the study DCR. The other patient was switched from TDF/FTC/EVG/c to DTG/ABC/3TC (Table 2, Patient 6) and historical genotypic testing revealed an M184V/I, M41L, T215Y and L74/I which predicted that DTG was the only fully active agent in the study DCR. Both patients had suboptimal adherence documented throughout the study period and both had genotypic testing performed at the time of DCR discontinuation and no new treatment-emergent RAMs were observed. Both patients were switched to a different DCR after discontinuation of study treatment, and both achieved HIV-1 RNA < 50 copies/mL on their new regimens (Table 2). Of the 5 patients on functional dual therapy, three had baseline HIV-1 RNA < 50 copies/mL, and two had baseline HIV-1 RNA ≥ 50 copies/mL. Two were switched from regimens containing ≥ 3 ARV drug classes and three were switched from regimens containing a dual NRTI plus boosted PI. All had baseline M184V/I, and 1 had additional NRTI RAMs. Three had suboptimal adherence documented throughout the study period whereas 2 had “100% adherence” documented at all visits. Three had genotypic testing performed shortly after discontinuation of study treatment and there was no evidence of new treatment-emergent RAMS in 3/3 patients. Two patients did not have genotypic testing performed despite persistent viremia on the study DCR. At the end of the study period, one viremic patient was lost to follow-up and 4/5 were switched to alternative regimens; in 3 of these patients the alternative regimen included DTG. All four patients achieved HIV-1 RNA < 50 copies/mL on their new regimens (Table 2).

**Table 2:** Clinical details and follow-up for patients with persistent HIV-1 RNA ≥ 50 copies/mL (N = 7)

	Regimen prior to DCR switch	Baseline DCR	Baseline RAMs	Active ARVs in DCR	HIV-1 RNA (cps/mL)	Suspected reason for non-response	Treatment-emergent RAMs	Date of DCR DC and new ARV regimen	HIV-1 RNA following DCR DC (cps/mL)
Patient 1	TDF/FTC/DRV/r/RAL	DTG + TDF/FTC	M184V/I	DTG + TDF	BL: < 50 W24: 50, 51 W48: 2598	Unclear, 100% compliance reported	No post-treatment GT	DC at W48, changed to ETR/DRV/r/DTG	W52: < 50 on new regimen
Patient 2	TDF/FTC/DRV/r/ETR	DTG + ABC/3TC	M184V/I	DTG + ABC	BL: < 50 W12: 250 W52:1023	Non-compliance	None, GT performed 9 months after W52	DC at W52, changed to ABC/3TC/DTG/DRV/r	W55: < 50 on new regimen
Patient 3	TDF/FTC/DRV/r	DTG/ABC/3TC	M184V/I	DTG + ABC	BL: < 50 W8: < 50 W28: < 50 W48: 80, 90	Unclear, 100% compliance reported	No post-treatment GT	DC at W48, switched to DTG/DRV/c	W56: < 50 on new regimen
Patient 4	TDF/FTC/DRV/r	DTG + TDF/FTC	M41L, M184V/I, T215Y	DTG + TDF	BL: 70 W8: 30 W16: 130 W32: 570 W48: 120	Non-compliance	None, GT performed 2 weeks after W48	DC at W48, switch to ETR/TDF/FTC/DRV/r	W53: < 50 on new regimen
Patient 5	ABC/3TC/DRV/r	DTG + RPV	M184V/I, E138E/K	DTG	BL: 170 W20: 320 W28: 130 W40: 150 W56: 600 W80: 90 W92: 120 W104: 210	Non-compliance	None, GT performed 16 months after W104	DC at W104, changed to DTG/DRV/c	W108: < 50 on new regimen
Patient 6	TDF/FTC/EVG/c	DTG + ABC/3TC	M184V/I, M41L, T215Y, L74L/I	DTG	BL: 280 W4: 90 W12: 90 W24: 110	Non-compliance	None, GT performed 4 weeks after W24	DC at W24, changed to DTG/DRV/r	W48: 60 W52: < 50 on new regimen
Patient 7	ABC/3TC/FPV/r	DTG + ABC/3TC	M184V/I	DTG + ABC	BL: 1400 W4: < 50 W36: 724, 801	Non-compliance	None, GT performed 2 weeks after W36	Regimen continued, LTFU after W36	LTFU after W36

*DCR* dolutegravir containing regimen, *RAM* resistance associated mutation, *ARV* antiretroviral, *DC* discontinuation, *DTG* dolutegravir, *TDF* tenofovir, *FTC* emtricitabine, *DRV* darunavir, *r* ritonavir, *RAL* raltegravir, *ABC* abacavir, *3TC* lamivudine, *EVG* elvitegravir, *c* cobicistat, *FPV* fosamprenavir, *RPV* rilpivirine, *BL* baseline, *W* week, *GT* genotype, *ETR* etravirine, *LTFU* lost to follow up

## Discussion

Data from randomized clinical trials has demonstrated that 6–10% of virologically suppressed patients switched to DTG monotherapy experienced VF, and of those 29–100% developed INSTI resistance [5–7]. Studies evaluating virologically suppressed patients switched to dual DTG-based therapy with 3TC and RPV demonstrated VF rates of 1–3% and zero patients developed treatment-emergent INSTI resistance [2, 4]. In the case of DTG/RPV, 54% of VFs developed resistance to RPV but not DTG [4]. In our cohort, 2/19 (11%) treated with DTG functional monotherapy and 5/20 (25%) treated with DTG functional dual therapy with a non-cytosine NA experienced persistent HIV-1 RNA ≥ 50 copies/mL. Of these, 5/7 had post-switch genotypes and none developed treatment emergent INSTI resistance (Table 2).

The most likely reasons for persistent viremia in our cohort included suboptimal adherence and the presence of significant baseline resistance. Of those with persistent viremia and documented non-adherence, two were on DTG functional monotherapy, and three were on DTG functional dual therapy with a non-cytosine NA. Of those on functional monotherapy, one was on DTG/RPV and had baseline E138E/K which reduces RPV susceptibility [20]. The other was on DTG/ABC/3TC and had baseline M184V/I, L74I, M41L and T215Y, the combination of which severely reduces susceptibility to 3TC and ABC [20]. In both patients, the presence of these baseline RAMs in combination with suboptimal adherence may have contributed to persistent viremia.

Among the three patients on functional dual therapy with persistent viremia and documented non-adherence, two were on DTG/ABC and only had baseline M184V/I whereas the other was on DTG/TDF and had baseline M184V/I, M41L and T215Y. In addition to reduced 3TC and FTC susceptibility, the M184V/I mutation is associated with low-level ABC resistance and may have contributed to persistent viremia in those on DTG/ABC [20]. In contrast, this mutation is associated with increased TDF susceptibility and the delay of treatment emergent TDF resistance [20]. However, in the patient on DTG/TDF this “hypersensitizing” effect may have been reduced by the presence of baseline M41L and T215Y which in combination are associated with low-to-intermediate-level TDF resistance and may have contributed to persistent viremia in this patient [20].

Nonetheless, 32/39 patients in our cohort, 22% of whom were viremic at baseline with similar patterns of baseline resistance (all with M184V/I and over half with ≥ 1 additional NRTI RAM) on DTG-based functional mono-or dual therapy achieved or maintained virologic suppression. All 32 suppressed patients had “100% adherence” documented throughout the study period and cumulatively these data suggest that the primary reason for persistent viremia in the patients discussed above was likely non-adherence. This is also supported by the fact that 4/5 non-adherent, persistently viremic patients had baseline HIV-1 RNA ≥ 50 copies/mL which likely indicates a history of non-adherence prior to study entry.

Post-switch genotypes were only available for the 5 non-adherent patients with persistent viremia, and 4 of these were obtained on DTG. We observed no treatment-emergent NRTI or INSTI resistance, and 3/5 patients subsequently went on to achieve HIV-1 RNA < 50 copies/mL on a different DCR after discontinuation of the study regimen. One patient achieved HIV-1 RNA < 50 copies/mL on a non-DCR and the other was lost to follow up (Table 2). Though based on a small sample, this observation reinforces the high genetic barrier to resistance of DTG and its forgiveness in the setting of non-adherence, even in patients with pre-existing ARV drug resistance.

Two persistently viremic patients were documented as 100% adherent, both were on functional dual therapy; one was on DTG/TDF and the other was on DTG/ABC. Both had baseline M184V/I without additional NRTI mutations. In the patient on DTG/ABC, the reduction in ABC susceptibility conferred by the M184V/I may have contributed to persistent viremia. However, in the other on DTG/TDF, this mutation is expected to increase TDF susceptibility and does not fully explain the inability to maintain virologic suppression. Baseline mutations may have contributed to persistent viremia in these cases; however, it is unknown how accurate their documented adherence patterns were and whether non-adherence may have also played a role.

In 32/39 patients on DTG-based functional mono-and dual therapy, HIV-1 RNA < 50 copies/mL was achieved and maintained throughout the study period. All patients received DCRs with ≥ 2 ARV drugs, and though baseline resistance testing predicted either functional mono- or dual therapy, we acknowledge the possibility of partial activity from other ARV drugs deemed not fully active. This may explain the high virologic response rates observed in our study and the lack of treatment emergent INSTI resistance in those with persistent viremia due to “protection” of DTG by these partially active agents.

Overall, these results deepen our knowledge about the efficacy of DTG when used in combination with 0–1 fully active ARV drugs. Given the increased global use of DTG in settings where resistance testing and complete ARV drug histories may be inaccessible, these data provide reassurance that most patients on DTG-based functional mono-or dual therapy will achieve virologic efficacy. This is especially relevant in LMICs where DTG is being used in second- and third-line ARV regimens to treat those failing first line NNRTI-based regimens. In July of 2018, the World Health Organization (WHO) published updated HIV treatment guidelines which recommended DTG in combination with an optimized NRTI backbone as a preferred option for PLWH in whom non-DTG based regimens were failing [9]. This guidance was based on data from the DAWNING study which demonstrated that DTG-based therapy was safer and more effective than a PI-based second-line regimen [21]. In this study which took place in 13 LMICs, 627 participants failing an NNRTI-based regimen with a mean baseline HIV-1 RNA log_10_ of 4.2 copies/mL were randomized to switch to an optimized dual NRTI backbone plus lopinavir/r versus DTG [21]. At baseline, half had a CD4^+^ T-cell count of < 200 cells/mm^3^ and 82% had a baseline M184V/I mutation plus ≥ 1 additional NRTI RAM. At Week 48, virologic suppression was observed in 84% treated with a DTG-based regimen versus 70%% treated with a lopinavir (LPV)/r-based regimen, and treatment-related adverse events occurred in 16% treated with DTG versus 38% treated with LPV/r [21]. Subgroup analyses based on number of active NRTIs in the regimen revealed that DTG-based therapy was superior to LPV/r regardless of whether the regimen contained 2 or < 2 active NRTIs [21]. In DAWNING, all participants underwent genotypic resistance testing at screening and results demonstrated that only 20% of participants received a study regimen containing 2 fully active NRTIs whereas 80% of participants were on regimens containing < 2 fully active NRTIs [21].

Our data reveals similar efficacy of DTG-based regimens in the setting of functional mono-or dual therapy which may be common in LMICs among patients failing a first-line regimen given the lack of access to resistance testing outside of a randomized clinical trial [10]. However, there are some key differences to note which may caution extrapolation of our results to these settings, 72% of our cohort was virologically suppressed prior to switch with only 28% having a baseline HIV-1 RNA ≥ 50 copies/mL, median baseline CD4^+^ T-cell count was 542 cells/mm^3^ which may be significantly higher than CD4^+^ T-cell counts among failing patients in LMICs, all patients in our cohort had Clade B HIV-1 virus whereas different HIV subtypes are more common globally and our study predominantly consisted of Caucasian men whereas international populations may contain greater numbers of women and individuals of Black or African descent. However, in both our study and the DAWNING trial, high adherence was reported among patients achieving virologic efficacy on DTG-based functional mono-or-dual therapy [21] and overall, these data suggest that optimal adherence may be an important contributor to the success of DCRs containing fewer fully active ARV drugs.

In our cohort, virologic suppression was observed among 82% of patients, all of whom had a historical M184V/I mutation and in the DAWNING study, 84% of those in the DTG arm achieved virologic suppression despite 71% of these patients having a baseline M184V/I. To date, several studies have evaluated the efficacy of DTG/3TC in the setting of pre-existing NRTI resistance including among those with an M184V/I mutation. A subgroup analysis of the TANGO study demonstrated no impact of archived NRTI resistance on virologic outcomes through Week 48 with 4/4 patients found to have an archived M184V/I mutation achieving virologic suppression [18]. In the ART-PRO study, virologic suppression with DTG/3TC was observed in 86% of patients with historical 3TC resistance versus 95% of patients without historical 3TC resistance through Week 96, p = 0.61 [17]. Similar results were seen in the Shall We Dance study which sought to assess the reproducibility of the TANGO results among a real-world diverse cohort of PLWH [15]. The study population was divided into two groups, one which would have the met the TANGO study criteria (TANGO group) and the other which would not have met the TANGO study criteria (non-TANGO group). Among the latter, 54% had a prior history of virologic failure and 10% had a documented M184V/I on the last genotype [15]. In the TANGO-group, there was a 99.2% probability of maintaining virologic suppression through Week 144 compared to a probability of 98.5% at Weeks 48 and 96 and 95.7% at Week 144 in the non-TANGO group, p = 0.189. There was no difference in results when stratifying based on presence of historical M184V/I or history of VF [15]. More recently, the LAMRES study has also demonstrated no difference in the probability of VF among those switching to DTG/3TC when stratifying for the presence versus absence of historical M184V/I through 2 years (9.2% vs. 4.4%, p = 0.345) [22]. Though use of DTG/3TC is not indicated among those with a history of M184V/I, these findings suggest that the presence of this mutation does not significantly impact virologic outcomes. This may be due to several factors which include the ability to suppress and maintain low viral loads on 3TC due to impaired viral fitness associated with the M184V/I mutation and the delay in emergence of additional drug resistance mutations associated with DTG in the presence of M184V/I as has been observed in vitro [23].

Among our study participants, 72% were suppressed at baseline prior to DCR switch however due to the retrospective nature of the analysis and implementation of a different electronic medical record (EMR) prior to study start, we were unable to accurately calculate duration of viral suppression for patients in our cohort. This has important implications as prior data has demonstrated that duration of viral suppression is associated with lower VF rates in patients with both previous treatment failure and HIV drug resistance [24–26]. The UK CHIC study conducted among 12, 648 treatment-experienced adults revealed that after 4 years of viral suppression, VF rates in patients with multiple prior treatment failures were similar to those in patients with no history of treatment failure [26]. Data from the REACH cohort which included 221 marginally housed PLWH demonstrated that the range of adherence required to sustain viral suppression was wider after 12 months of continuous viral suppression versus 1 month [24]. A study conducted by the ARCA collaborative group evaluated the impact of NRTI resistance on the probability of VF in patients switched to a dual NRTI plus DTG. Findings showed no impact of previous NRTI resistance on the risk of VF and that longer durations of viral suppression prior to switch were associated with a lower risk of VF [25]. These data suggest that duration of viral suppression prior to DCR switch in our cohort may have contributed to virologic efficacy among those with baseline HIV-1 RNA suppression. This hypothesis deserves further exploration; if a minimum time of viral suppression which significantly lowers VF risk can be established, there may be a reduced requirement for baseline genotypic testing prior to switching to a DTG-based regimen which may be of benefit in settings where access to historical and prospective resistance testing is limited.

Limitations of this study include a small sample size, the retrospective nature of the analysis, the lack of control group, possible inaccuracy of documented information, and that data are from a single center in the Southeastern United states. Our cohort also predominantly consisted of Caucasian men which limits generalizability to other populations living with HIV and implementation of the current electronic medical record (EMR) version occurred in January of 2013, hence some data prior to this date were not readily available. Though historical genotype results had been scanned in for all patients, we did not have access to many other historical laboratory values which limited our ability to describe certain characteristics that may have impacted interpretation of our study results.

Despite these limitations, this is the first report of virologic outcomes of patients treated with DTG-based functional mono-and-dual therapy from a US cohort and provides valuable insight into the efficacy and barrier to resistance of DTG-based treatment strategies with fewer ARV drugs.

## Data Availability

Not applicable.
